# Additive-Free Gelatine-Based Devices for Chondral Tissue Regeneration: Shaping Process Comparison among Mould Casting and Three-Dimensional Printing

**DOI:** 10.3390/polym14051036

**Published:** 2022-03-04

**Authors:** Margherita Montanari, Alex Sangiorgi, Elisabetta Campodoni, Giada Bassi, Davide Gardini, Monica Montesi, Silvia Panseri, Alessandra Sanson, Anna Tampieri, Monica Sandri

**Affiliations:** Institute of Science and Technology for Ceramics, National Research Council (CNR), 48018 Faenza, Italy; alex.sangiorgi@istec.cnr.it (A.S.); elisabetta.campodoni@istec.cnr.it (E.C.); giada.bassi@istec.cnr.it (G.B.); davide.gardini@istec.cnr.it (D.G.); monica.montesi@istec.cnr.it (M.M.); silvia.panseri@istec.cnr.it (S.P.); alessandra.sanson@istec.cnr.it (A.S.); anna.tampieri@istec.cnr.it (A.T.)

**Keywords:** mould casting, 3D printing, biopolymers, tissue engineering, chondral regeneration, cartilage

## Abstract

Gelatine is a well-known and extensively studied biopolymer, widely used in recent decades to create biomaterials in many different ways, exploiting its molecular resemblance with collagen, the main constituent of the extra-cellular matrix, from which it is derived. Many have employed this biopolymer in tissue engineering and chemically modified (e.g., gelatin methacryloyl) or blended it with other polymers (e.g., alginate) to modulate or increase its performances and printability. Nevertheless, little is reported about its use as a stand-alone material. Moreover, despite the fact that multiple works have been reported on the realization of mould-casted and three-dimensional printed scaffolds in tissue engineering, a clear comparison among these two shaping processes, towards a comparable workflow starting from the same material, has never been published. Herein, we report the use of gelatine as stand-alone material, not modified, blended, or admixed to be processed or crosslinked, for the realization of suitable scaffolds for tissue engineering, towards the two previously mentioned shaping processes. To make the comparison reliable, the same pre-process (e.g., the gelatin solution preparation) and post-process (e.g., freeze-drying and crosslinking) steps were applied. In this study, gelatine solution was firstly rheologically characterized to find a formulation suitable for being processed with both the shaping processes selected. The realized scaffolds were then morphologically, phisico-chemically, mechanically, and biologically characterized to determine and compare their performances. Despite the fact that the same starting material was employed, as well as the same pre- and post-process steps, the two groups resulted, for most aspects, in diametrically opposed characteristics. The mould-casted scaffolds that resulted were characterized by small, little-interconnected, and random porosity, high resistance to compression and slow cell colonization, while the three-dimensional printed scaffolds displayed big, well-interconnected, and geometrically defined porosity, high elasticity and recover ability after compression, as well as fast and deep cell colonization.

## 1. Introduction

The spread of cartilage injuries, combined with their often-asymptomatic origin and the lack of intrinsic chondral tissue healing capacity, makes chondral regeneration a significant clinical challenge [1,2,3,4]. Many are key points to consider when designing a tissue-to-be capable of revealing full biocompatibility, gradual resorbability, suitable mechanical properties, and fine control of the inner and outer geometry resulting in a proper porosity [5,6]. It has been proven how a thorough design of the material composition and geometry can deeply influence the final device properties, even arriving to lead and govern cells’ behavior only by means of the mechanical and geometrical stimuli provided to them [7]. To this end, being able to design and develop a directly implantable biomaterial capable of in situ, elegantly direct cell differentiation and growth in virtue of its mere composition, geometric structure, and mechanical properties is of great appeal [6]. In this context, three-dimensional printing has recently been pointed out as a step forward, compared to the well-known and commonly used mould casting technique, towards a better design and control of the desired regenerative material [5,8,9].

As reported by Ligon et al. [10], conventional techniques of scaffold fabrication such as phase inversion, solvent casting, solute leaching, and electrospinning often lack the ability to incorporate fine internal architectural details and controlled porosity while ensuring reproducibility. Especially in tissue engineering, where the need for patient-specific medical devices is increasing, 3D printing looms as an interesting option by addressing these issues through a precise regulation of pore sizes and their uniform distribution throughout the scaffold [3,10,11,12,13,14,15,16,17,18].

Nevertheless, together with many advantages allowed by this technology, such as precise control of the outer shape and inner geometry, come different challenging aspects that need to be faced and overcome. Among these, the operating conditions (such as nozzle diameter, extrusion rate, printing temperature) together with material-related factors such as gel rheology [19,20,21,22,23]. For load-bearing tissues, it is important to understand the correlations between structure and function to define tissue engineering design criteria. The viscoelastic property of cartilage derives from its extracellular matrix (ECM) composition that is made of water, collagen, and glycosaminoglycans [24,25,26,27,28,29,30].

Starting from considering the above-mentioned features but keeping in mind the ongoing difficulties related to collagen printing, mainly due to its fibrous nature, we decided to opt for printing gelatine (Gel), a collagen-derived biopolymer with proven biocompatibility [31], trying to process it as stand-alone material, i.e., without the addiction of any additive or crosslinker, nor during the processing nor post-processing steps.

Within this work we aim to provide a lacking comparison between two of the main employed shaping processes in tissue engineering, 3D printing, and mould casting, employing the same starting material, Gel. Gel was chosen among all the possibilities thanks to its collagen-derived nature and less organized macro-molecular structure, which make it suitable for printing without losing its biocompatibility and resorbability [32,33,34]. Moreover, whereas it can be efficiently obtained from food waste, it results in an extremely low-cost raw material, easily convertible into a value-added product, following the principles of the circular economy [31]. A comparison in terms of feasibility of the process with the employed material and outcomes in terms of morphological, chemico-physical, mechanical, and biological performances of the obtained scaffold was performed and the results discussed in the light of their possible application.

## 2. Materials and Methods

### 2.1. Gel Solution

**Scheme 1:**
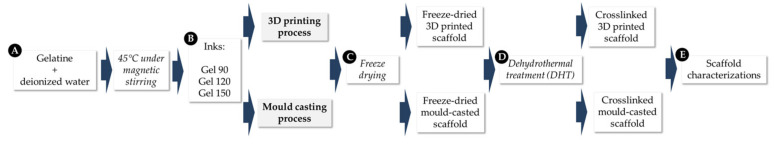
Schematic representation of the processes’ comparison design and realization, starting from the gelatin solution preparation (**A**), passing by the different concentrations’ ink realization (**B**), the two different processes, respectively, 3D printing and mould casting up to the final scaffolds, first freeze-dried (**C**) and then crosslinked (**D**) to make them stable and ready to be further characterized (**E**).

Gelatine (Gel) powder with mesh 4 and bloom 280, extracted from pig skin, was received by Italgelatine (Cuneo, Italy). Gel aqueous solutions at different concentrations were prepared by dissolving Gel powder in ultra-pure water (0.055 µS/cm, 25 °C) at 45 °C under strong and constant magnetic stirring, until perfectly homogeneous and transparent solutions were obtained (Scheme 1). In particular, for the 3D printing, three different Gel aqueous solutions of 90 mg/mL (Gel90), 120 mg/mL (Gel120) and 150 mg/mL (Gel150) were prepared. To make the 3D printed scaffold comparable with the mould casted, the concentrations of the seconds were adjusted considering the infill rate of the 3D-printed scaffold to which they were going to be compared, as reported in Table 1.

### 2.2. Rheology

The rheological behaviour of the Gel solutions was investigated at 45 °C by measuring their viscosity with a ramp test in controlled stress mode and at 25 °C, when they were in the gel state, by measuring their viscoelastic properties with small amplitude oscillation shear (SAOS) tests. All the measurements were performed with a rotational rheometer (C-VOR 120, Bohlin Instruments, Cirencester, UK). The ramp test was performed using a cone/plate CP4/40 (Ø = 40 mm, conicity = 4°) geometry by increasing the shear stress from 0.05 Pa up to 10 Pa with a sweep time of 300 s. Before starting the test, the samples were allowed to rest for 3 min to reduce the possible influence on the measurement of the solutions’ handling. A solvent trap was used to avoid water evaporation during the test. Amplitude and frequency sweep tests were carried out using a plate/plate PP20 (Ø = 20 mm) geometry with a large gap of 6 mm. The amplitude sweep tests were conducted by applying stresses ranging from 0.1 Pa up to 5.73·10^4^ Pa at the frequency of 1 Hz, while the frequency sweep test was conducted by decreasing the frequency from 10 to 0.01 Hz with an applied constant stress of 5 Pa that, in each case, resulted in being inside the linear viscoelastic region (LVER). Paraffin oil with low viscosity (Carlo Erba, Milan, Italy) was used to prevent the samples from drying out during the viscoelastic measurements.

### 2.3. Scaffold Shaping Processes

Mould-casted scaffolds were obtained pouring the obtained Gel solutions in Ø = 6 mm and h = 6 mm polytetrafluoroethylene (PTFE) moulds that were then freeze-casted. The freeze casting process consisted of a pre-freezing step with a controlled freezing ramp of 50 °C/h down to −40 °C, followed by two heating ramps, of 5 °C/h from −40 °C to −5 °C and of 3 °C/h up to 20 °C, respectively. The whole process was carried out under vacuum (P = 0.086 mbar). Finally, the scaffolds were crosslinked by means of a dehydrothermal (DHT) treatment that, as per the literature, was performed for 48 h in a vacuum oven at 160 °C with a pressure of 0.01 mbar [32,35,36,37,38,39,40].

The 3D scaffolds design was performed by using a dedicated parametric three-dimensional drawing and design software. Then, to produce the real objects, a slicing software was used. 3D-printed scaffolds were obtained by employing the Gel solutions as inks for a screw extrusion-based 3D printer (DeltaWasp 2040, Wasp, Massa Lombarda, Italy). Each solution was poured, at 45 °C, in an 81 mL (Ø = 4 cm; h = 6.5 cm) steel sink and allowed to cool down to room temperature overnight to allow the complete gelation. The whole extrusion process was carried out at room temperature (25 °C) at 3.5 bar pressure. For the scaffolds’ realization, the 3D printer was equipped with a Ø = 0.41 mm conic nozzle. The as-produced 3D printed scaffolds were then freeze-dried and crosslinked by DHT treatment following the same protocols previously reported for the mould-casted scaffolds and next punched to obtain 6 mm diameter and 6 mm height cylindrical samples.

### 2.4. Morphological Characterization

Scaffolds morphology was evaluated through environmental scanning electron microscopy (ESEM, Quanta 600 FEG, FEI Company, Hillsboro, OR, USA) and digital optical microscopy (Hirox RH-2000, 3D Digital Microscope, Hirox Europe, Limonest, France). The samples for ESEM observations were Au coated by sputtering (QT150T, Quorum Technologies Ltd., Lewes, UK).

The scaffolds’ total and macro porosities, as well as the swelling and degradation tests, were assessed by means of density-based and water-squeezing methods, respectively, as reported by Campodoni et al. and Gostynka et al. [33,41]. Briefly, the apparent density (*ρ_a_*) of the cylindrical scaffolds was calculated through the equation:

(1) $$\rho_{a}=\frac{W}{\pi \times {\left(\frac{D}{2}\right)}^{2}\times H}$$

where *W* is the dry scaffold weight, *D* the diameter and *H* the height. The obtained *ρ_a_* was then divided by the material theoretical density (*ρ_t_*) to obtain the total porosity:

(2) $$Total\;porosity_{\%}=100-\left(\frac{\rho_{a}}{\rho_{t}}\times 100\right)$$

With a replicate number of three (*n* = 3) and values expressed as mean ± standard error.

The macro-porosity was instead calculated by the equation:

(3) $$Macropores\;volume_{\%}=\frac{W_{swollen}-W_{squeezed}}{W_{swollen}}\times 100$$

To perform the test, scaffolds were soaked in phosphate buffered saline (PBS) (Sigma Aldrich, Saint Louis, MO, USA) at 37 °C for 1 h before being weighed (*W_swollen_*), squeezed and weighed again (*W_squeezed_*).

### 2.5. Chemico-Physical Characterization

Swelling test [33,41,42] was performed by soaking the samples in PBS solution with 0.1% *w*/*v* of NaN_3_ at 37 °C by shaking and weighing the swollen samples at different times (0.5; 1.5; 3; 6; 24; 48 h). The swelling ratio (*S_r_*) was calculated as:

(4) $$S_{r}=\frac{W_{swallen}-W}{W}$$

where *W_swallen_* was the swollen sample weight and *W* the dry sample weight before the soaking.

Degradation [33] test was performed at 7 and 21 days. The samples were treated the same as for the swelling test; however, on timepoints they were washed, and lyophilized Samples were then weighed and their degradation ratio (*D_%_*) calculated as:

(5) $$D_{\%}=\frac{W-W_{f}}{W_{f}}\times 100$$

where *W* was the dried sample initial weight and *W_f_* was the freeze-dried sample weight at a specific time point.

### 2.6. Mechanical Characterization

The scaffolds’ mechanical properties were evaluated by using a dynamic mechanical analyzer Q800 (TA Instruments, New Castle, DE, USA in uniaxial submersion-compression mode. Measurements were carried out in submersion in PBS at 37 °C and tests performed after the sample’s overnight immersion in PBS at 37 °C under shaking.

The Young’s modulus was evaluated by means of a stress–strain test [33,41] consisting in an isothermal period of 5 min at 37 °C, followed by a force ramp rate of 0.1 N/min from 0 N to 1 N, then a force ramp rate of 0.5 N/min from 1 N to 5 N to the end with a force ramp rate of 1 N/min from 5 N to 18 N.

A creep test [33,41] was carried out to evaluate the recovery ability of the samples. The analysis was performed at 37 °C in submersion, with a creep time of 15 min, a recovery time of 15 min, and an applied stress of 6·10^−2^ MPa.

The viscoelastic behavior of the scaffold was assessed by means of a multifrequency test [33], performed in submersion at 37 °C in a frequency range from 1 to 10 Hz and at a constant amplitude of 75 µm (*n* = 5).

### 2.7. Preliminary Biological Study

Human Chondrocytes cell line (CHON-002) purchased from American Type Culture Collection (ATCC^®^ CRL™-2847, Manassas, VA, USA) were cultured in standard medium composed by Dulbecco’s Modified Eagle Medium/F-12 Nutrient Mixture (DMEM/F-12, Gibco, Waltham, MA, USA) with Glutamine (GlutaMAX, Gibco, Waltham, MA, USA), supplemented with 10% Foetal Bovine Serum (FBS) and 0.1 mg/mL G418 Sulphate (50 mg/mL) (Gibco, Waltham, MA, USA). The cultures were kept in an incubator at 37 °C, in a 5% CO_2_ atmosphere, and in a controlled humidity conditions. The cells were detached from culture flasks by trypsinization and centrifugated. The cell number and viability were defined using the Trypan Blue Dye Exclusion test.

Both Gel120 f70 mould-casted and 3D-printed scaffolds (of 6 mm height and 6 mm diameter and sterilized by γ-ray irradiation (25 kGy)) were pre-soaked in culture medium for 24 h before seeding ~2.5·10^4^ cells/scaffold by carefully dropping 20 µL of cell suspension on each scaffold upper surface. After allowing cell adhesion for 30 min, 1.5 mL/well of culture medium was added to the scaffolds and gently changed every 2–3 days. The cultures were kept for 7 days at 37 °C with 5% CO_2_ atmosphere and controlled humidity conditions. All cell handling procedures were performed under laminar flow hood and in sterile conditions.

Quantitative cell viability and proliferation analysis was performed via MTT Assay [43,44]. In detail, MTT reagent [3-(4,5-dimethylthiazol-2-yl)-2,5-diphenyltetrazolium bromide] was firstly dissolved in PBS 1X (5 mg/mL). The scaffolds were incubated with 10% well-volume MTT solution for 2 h at 37 °C and 5% CO_2_ atmosphere conditions. Later, each scaffold was incubated with dimethyl sulfoxide for 15 min. In this assay, the metabolically active cells react with the tetrazolium salt in the MTT reagent to produce a formazan dye that can be observed at λ_max_ of 570 nm, using a Multiskan FC Microplate Photometer (Thermo Fisher Scientific, Waltham, MA, USA). This absorbance is directly proportional to the number of metabolically active cells. For each type of material, two scaffolds were analyzed at each time point (1, 3 and 7 days of culture). The results were expressed as Mean ± Standard Error of the Mean plotted on the graph. Statistical analysis was conducted by two-way ANOVA analysis of variance, followed by Sidak’s multiple comparisons test by the GraphPad Prism software (version 6.0), with statistical significance set at *p* ≤ 0.05.

Qualitative cell viability was analyzed by Live/Dead assay kit (Invitrogen, Waltham, MA, USA) according to the manufacturer’s instructions [43,45]. Briefly, the samples were washed with PBS 1X for 5 min and incubated with Calceinacetoxymethyl (Calcein AM) 2 µM plus Ethidium homodimer-1 (EthD-1) 4 µM for 15 min at 37 °C in the dark. The samples were then rinsed in PBS 1X. Images were acquired by an inverted Nikon Ti-E fluorescence microscope (Nikon, Tokyo, Japan). One sample per group was analyzed at day 1 and 3.

An additional qualitative analysis to highlight the scaffold colonization by the cells was performed by cell nuclei detection. Briefly, the scaffolds were washed in PBS 1X for 5 min and fixed in 4% (*w*/*v*) paraformaldehyde (PFA) (Sigma, Tokyo, Japan) for 15 min. 4′-6-Diamidino-2-phenylindole (DAPI) (Invitrogen, Waltham, MA, USA, 300 µM) staining was performed to highlight cell nuclei [46]. The samples were visualized with inverted Ti-E fluorescence microscope (Nikon, Tokyo, Japan). One sample per group was analyzed at day 1, 3, and 7.

## 3. Results and Discussion

### 3.1. Rheology of the Gel Solutions

Considering their potential printability features (e.g., good extrudability, acceptable filament swelling, shape maintenance, etc.), three different Gel concentrations (90, 120, and 150 mg/mL) were initially chosen for the realization of the 3D-printed scaffolds, then adapted for the mould casting, as described in Table 1.

The rheological properties of 3D printing inks are crucial for the success of the process. They affect the printability, performance, and results of the print [47,48].

**Figure 1:**
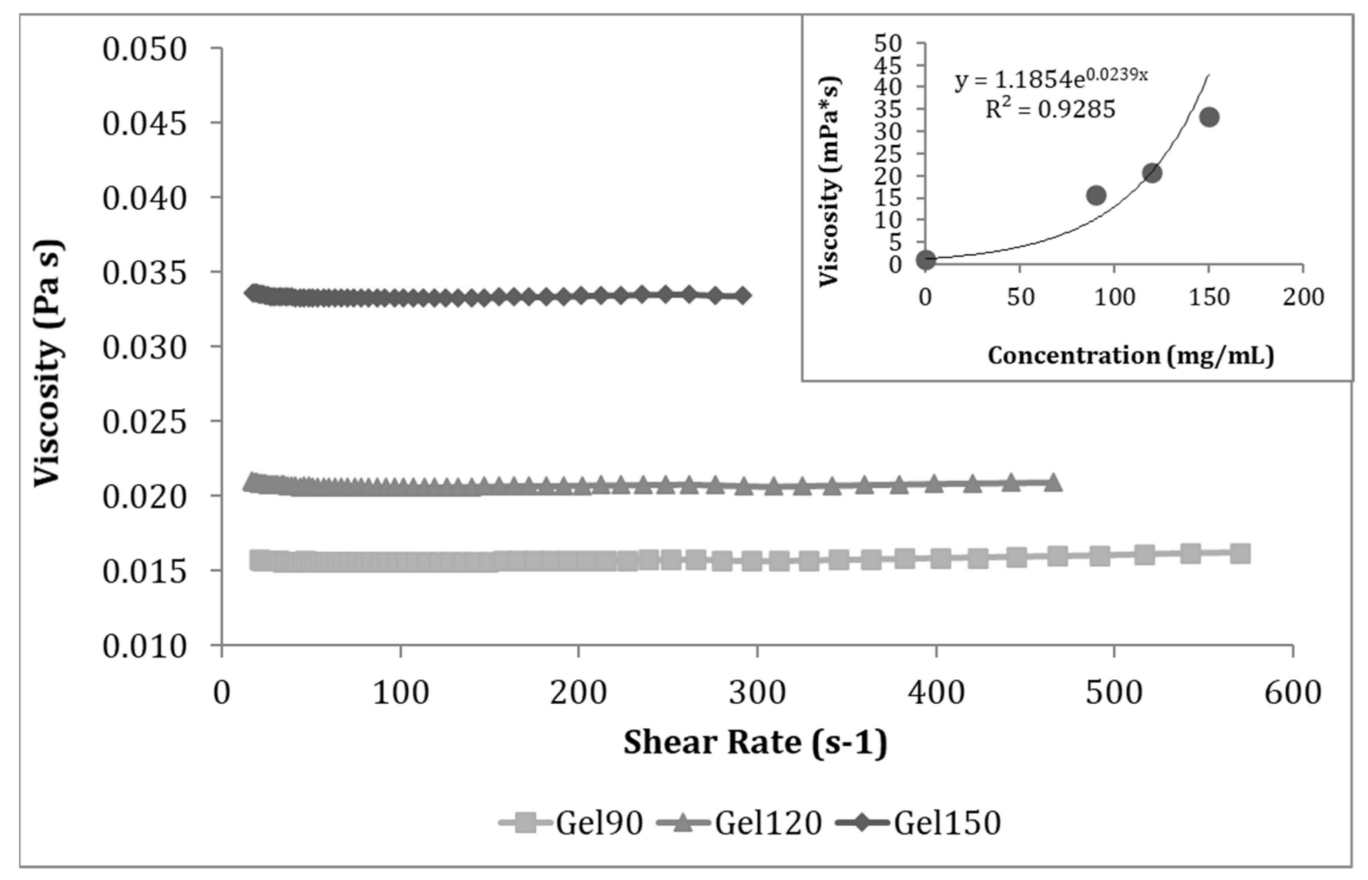
Demonstration of the three different Gel solutions Newtonian behaviour, tested at 45 °C; in the inset is shown the non-linear increase of viscosity with the concentration.

Through the viscosity test performed at 45 °C, the Newtonian behavior of all the Gel solutions was assessed. Moreover, a substantial increment in the Gel150 dynamic viscosity in respect to Gel90 and Gel120 was recorded (Figure 1). The dynamic viscosity increased from 15.7 mPa s for Gel90, to 20.7 mPa s for Gel120, and to 33.4 mPa s for Gel150, with a non-linear trend with respect to concentration (see inset in Figure 1).

**Figure 2:**
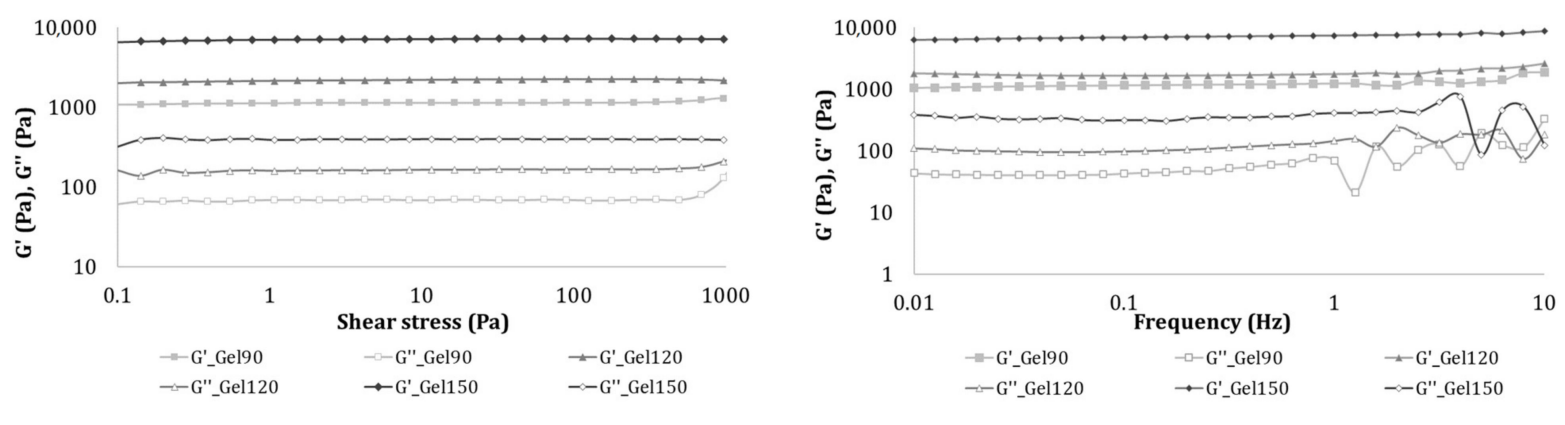
Amplitude stress sweep tests at 1 Hz (**left**) and frequency sweep tests (**right**) for the three gels at 25 °C.

The amplitude sweep test and the frequency sweep test (Figure 2) were performed on the solid hydrogels at 25 °C in order to evaluate their complex modulus G*, meaning that the overall resistance to deformation of the material, given by the combination of its recoverable (elastic G′) and non-recoverable (viscous G″), that could be described as (G* = G′ + iG″) and loss angles. The amplitude stress sweep test at 1 Hz shows that all the three gels remained in the linear viscoelastic region up to almost 500 Pa. For each Gel, the elastic modulus was almost an order of magnitude higher than the viscous one, and their values increased with the concentration of gelatin, consistent with the viscosity. For the most concentrated sample (Gel150) the elastic modulus was about 7 kPa, resulting in a value excessively high for the 3D printing process, while the value of 2.2 kPa of Gel120 and of 1.5 kPa of Gel90 resulted in them being suitable for the application.

To be conservative, the frequency sweep test was conducted keeping the shear stress fixed at 5 Pa for all the frequencies investigated. Such test shows that the two moduli are almost constant with the frequency, as is expected for hydrogels. In addition, they allowed us to confirm the prevalence of the elastic component on the viscous one of the Gel solutions in the gel state and to assess a non-linear dependence of viscosity and G* values on Gel solutions concentration. Indeed, to a linear increment of the gel concentration from Gel90 to Gel150, a significant higher increase in viscosity and G* values passing from Gel120 to Gel150 was recorded, resulting in strongly different performances in terms of extrusion. Based on the rheology results and after a preliminary extrusion test on the obtained Gel solutions, Gel150 was ruled out from further experiments due to its extremely difficult printability, in line with its rheological behavior; thus, only Gel90 and Gel120 were used in the scaffold shaping processes.

Passing from 90 to 120 mg/mL of polymer concentration, the increase of viscosity was essentially due to the increased interaction between the polymer macromolecules and solvent. Meanwhile, passing from 120 to 150 mg/mL, the interactions among the polymeric chains became relevant with an increasing overlapping of the macromolecules, even if, probably, no entanglement occurs at those concentrations [49].

### 3.2. Scaffolds Shaping Processes

Mould-casted scaffolds were obtained by pouring the as-obtained Gel solutions in Ø = 6 mm PTFE moulds, then freeze-casted. PTFE was chosen among all the possible materials thanks to its extremely low friction coefficient, non-reactive nature, and good thermal conductivity [50] in order to allow a good directional freeze-casting of the Gel solutions, not influenced by any possible interaction with the mould, nor chemical or physical, and with an effective conduction of the heat exchange during the whole process. Strictly controlling the freezing and heating ramps’ rate and the time of freeze casting made it possible to control and direct the size, shape, and orientation of the ice crystals, meant to be the pores within the material, thus controlling the final scaffold morphology [32,35,36,40]. The whole freeze casting process of the Gel mould-casted scaffolds was designed to obtain a final structure characterized by ordered longitudinal aligned pores.

Since the realization of a 3D printing scaffold made just of gelatine, without any extra crosslinker or additive, requires the printing of full borders around the main geometrical structure to support and anchor it, we opted for printing a Ø = 35 mm and h = 10 mm cylindrical scaffold from which Ø = 6 mm and h = 6 mm cylindrical scaffolds were carefully cut out. This approach, aimed at excluding the border contribution, was pursued to better compare the mesh, infill rate, and Gel solution concentration of the 3D samples versus the corresponding mould-casted same-sized samples.

For the printing process, a 25 °C printing temperature, combined with an extrusion pressure of 3.5 bar and a Ø = 0.41 mm tip, was selected as the best conditions, since it was able to combine a precise, constant, and continuous material flow, shape maintenance, and model fidelity with an extremely simple and low-cost process. Higher printing temperatures resulted in a total loss of shape maintenance, while higher extrusion pressures resulted in an excessive swelling of the printing filament with a consequent strong loss in model fidelity. A tip of Ø = 0.41 mm was selected as the best dimension to ensure high resolution printing while dealing with all the previously cited parameters. Different infill percentages, ranging from 50 to 70%, with a reticuled mesh were chosen to be further investigated. From some preliminary tests (data not shown), going down to less than 50% of infill was indeed proven to be useless, since the pore dimension turned out to be excessive for promoting any cell colonization, while, on the contrary, going up to more than 70% of infill was proven to be pointless since the model fidelity would be extremely decreased after freeze-casting and because of the excessive closeness of the filaments (data not shown). The 3D printed scaffolds were then freeze casted at the same conditions as the previously reported mould-casted ones.

To improve the chemical stability and preserve the mechanical performance of the produced scaffolds in wet conditions, a physical crosslinking procedure, named dehydrothermal (DHT) treatment, was selected. This was carried out to improve their behavior in physiological environments without adding any extra components, and thus variables, to the pure polymer. DHT treatment is widely implied in the reticulation of collagen and collagen-derivatives such as gelatin, with demonstrable improvements to their stability and mechanical properties, if well balanced with the treatment-related polymer degradation unavoidably occurring at the pressure and temperature required for the successful crosslinking of the material [37,38,39].

### 3.3. Morphological Characterization

**Figure 3:**
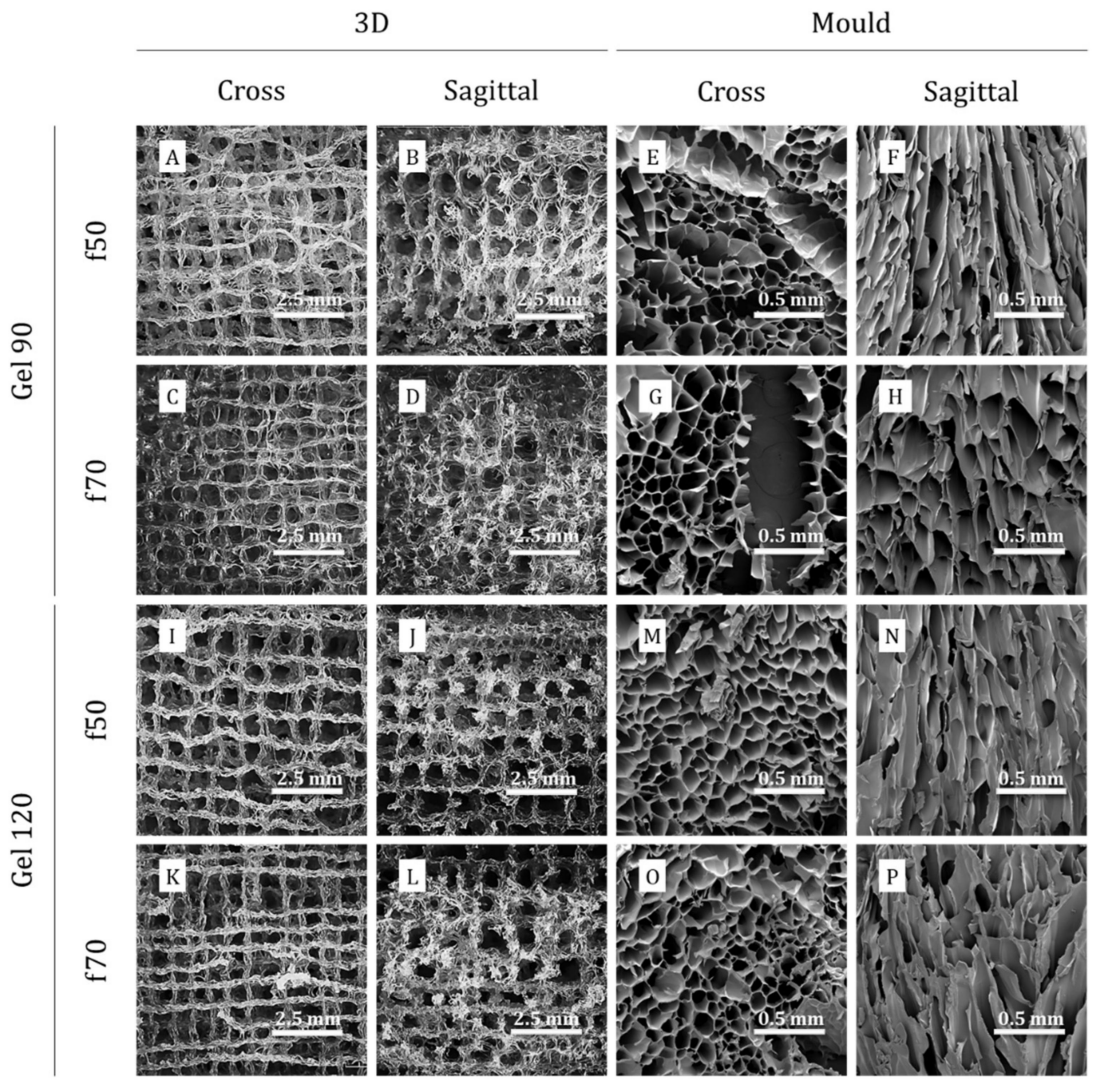
Cross and sagittal section of 3D printed and mould-casted scaffolds, after freeze casting, respectively, acquired by optical microscope and ESEM. (**A**–**D**,**I**–**L**) morphology of 3D printed scaffolds acquired by optical microscope; (**E**–**H**,**M**–**P**) morphology of mould casted scaffolds acquired by ESEM.

Morphological characterizations were performed on the resulting scaffolds after freeze-casting and DHT, aiming to determine the outcomes of the two different shaping processes. Due to the different dimensions of their most relevant morphological features, such as pores shape and dimension, two different characterizations techniques were used to highlight them: ESEM for the mould-casted samples and optical microscopy for the 3D-printed ones (Figure 3).

What was immediately clear from the morphological characterization was that the directional mould casting process, performed to obtain well-aligned and ordered porosity, was successful. As it is clearly observable from both the cross and the sagittal section of the mould-casted samples, there were no relevant differences among the concentrations used (Figure 3). The moulds obtained by Gel90 were, nevertheless, presenting significant and extensive cracks (Figure 3). Moreover, it is visible that, while for the mould-casted scaffolds there were no differences in the pores dimensions or geometry depending on the Gel solution concentration, that was not the same for the 3D-printed scaffolds, for which a higher Gel solution concentration resulted in a better shape maintenance and model fidelity, especially at higher infill percentages. This was probably ascribable, as for the cracks in the mould-casted scaffolds, to the higher water amount in Gel90, which introduced a strong distortion effect on the sample during the freeze-casting process. 3D Gel90 f70 resulted in scaffolds with smaller and round-shaped pores, likely due to the mesh lines touching and merging before being stabilized in the freeze-casted structure.

**Figure 4:**
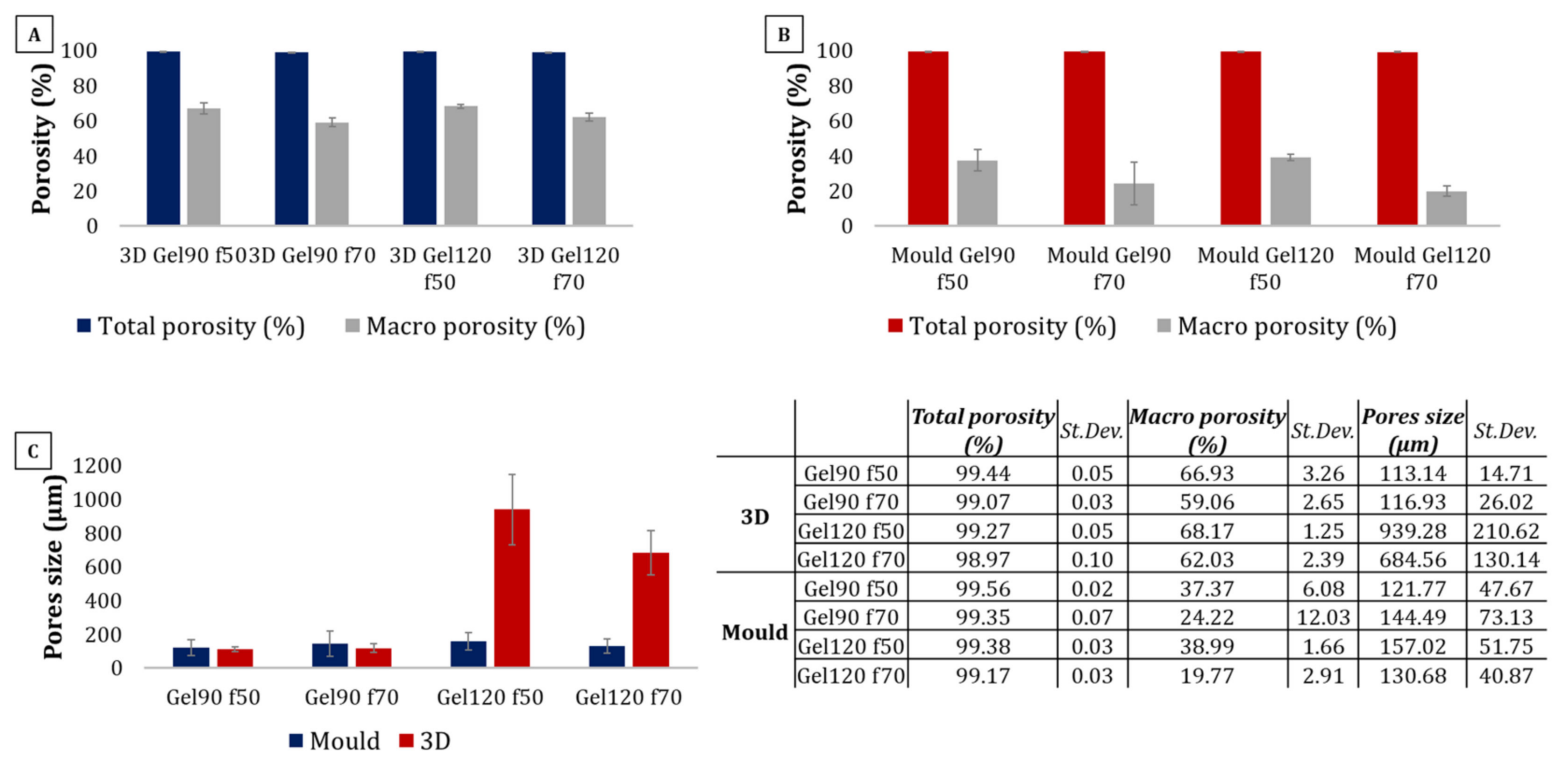
Total and macro porosities of 3D-printed (**A**) and mould-casted (**B**) scaffolds, and their corresponding pore sizes (**C**).

The above-mentioned morphological features emerged also in the porosity size evaluation performed by images analysis (Figure 4C), in which the mould-casted scaffolds showed no significant differences in pore size at any conditions (from 121.77 µm ± 47.67 of Gel90 f50 to 157.02 µm ± 51.75 of Gel120 f50); on the other hand, Gel90 samples showed a substantially more similar porosity to the mould-casted scaffolds than to the Gel120-printed ones, with the same printing model used (with a porosity of 113.93 µm ± 14.71 for Gel90 f50 and 116.93 µm ± 26.02 for Gel90 f70 versus 939.28 µm ± 210.62 for Gel120 f50 and 684.56 µm ± 130.14 for Gel120 f70). The Gel120-printed scaffolds’ porosity was instead consistent with the one designed and expected, with a bigger porosity for Gel120 f50 and a reduced one for Gel120 f70 (Figure 4C).

Density method [33] allowed the total porosity of the realized scaffolds to be calculated, which resulted in almost 99%, independently from the process used (Figure 4A,B). The water-squeezing method [33,41] was employed to estimate the macro-porosity of the scaffolds, which turned out to be almost double for the 3D-printed scaffolds (Figure 4A,B) thanks to their better pores geometry and interconnection, as proven also by the morphological characterization. Both processes allowed, therefore, the highly porous scaffold crucial for proper cell colonization to be obtained, anchoring and spreading also for their nourishment, waste collection, and expulsion. Nevertheless, while with the mould casting process the porosity turned out to be random, mostly small, and anisotropic, with the 3D printing the resulting scaffold showed a good model fidelity, with a controllable and tailorable isotropic porosity of one order of magnitude higher, a feature that can be of great relevance for in vivo tissue development.

### 3.4. Chemical-Physical Characterization

**Figure 5:**
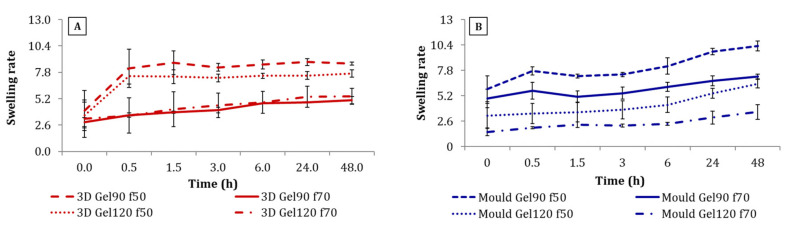
Swelling ratios for 3D-printed (**A**) and mould-casted (**B**) scaffolds represented as mean with st.dev. bars.

The swelling test [33,42] demonstrated a clear difference between the 3D-printed and mould-casted scaffolds (Figure 5A,B). For the 3D-printed ones, the infill rate emerged as the most relevant parameter affecting the swelling, with f50 showing a swelling ratio significantly higher than the f70, while for the mould-casted samples the concentration of the Gel solution (Table 1) is the determining parameter, with a gradual but significant swelling ratio decrease from Gel90 f50 to Gel120 f70. For the 3D-printed scaffolds, this resulted in consistency with their building process and resulting morphology, while the different behavior of the mould casted ones can be arguably ascribable to the reduction of water with the solution concentration increment. This makes, indeed, the growth of long longitudinal-aligned crystals during the freeze-casting process, generating a sparingly interconnected porosity, less likely to efficiently absorb the medium.

**Figure 6:**
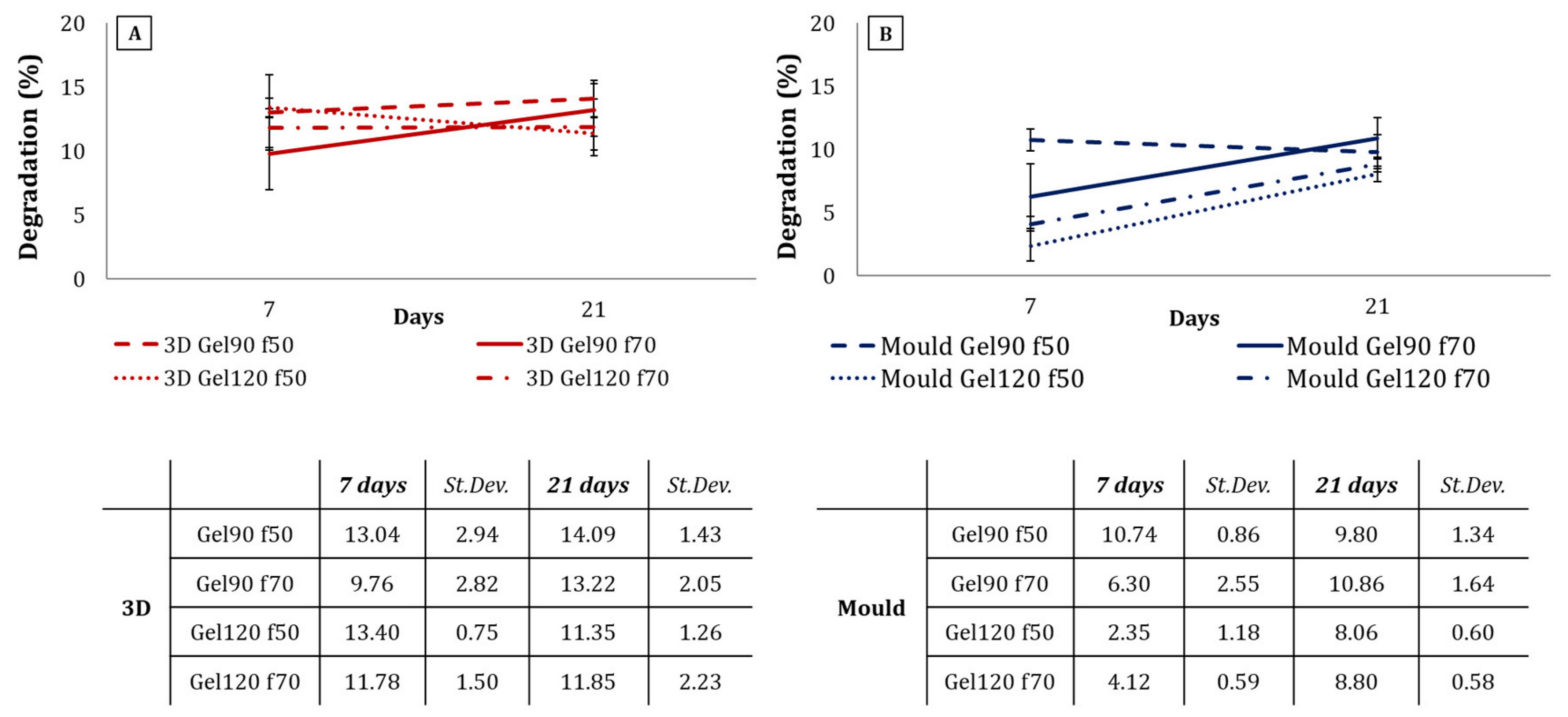
Degradation of 3D-printed and mould-casted scaffolds at 7 and 21 days, represented as mean with st.dev. bars. (**A**) degradation of 3D printed scaffolds; (**B**) degradation of mould casted scaffolds.

The degradation test [33] was performed to evaluate the suitability of the realized scaffolds to be implied in the physiological environment, adequately withstanding the cellular colonization and growth before starting to degrade to be resorbed. Both the casted and printed scaffolds proved good performers, with degradation ratios under 15% at 21 days, nearly constant from 7 to 21 days for the most of the 3D-printed scaffolds (Figure 6A,B). The slightly higher degradation ratio of the 3D-printed ones (Figure 6A) is likely attributable to their higher macro-porosity and better porosity interconnection, allowing the fluids to better flow through the scaffolds’ structure, fostering matter exchange with the medium.

### 3.5. Dynamic Mechanical Analysis

**Figure 7:**
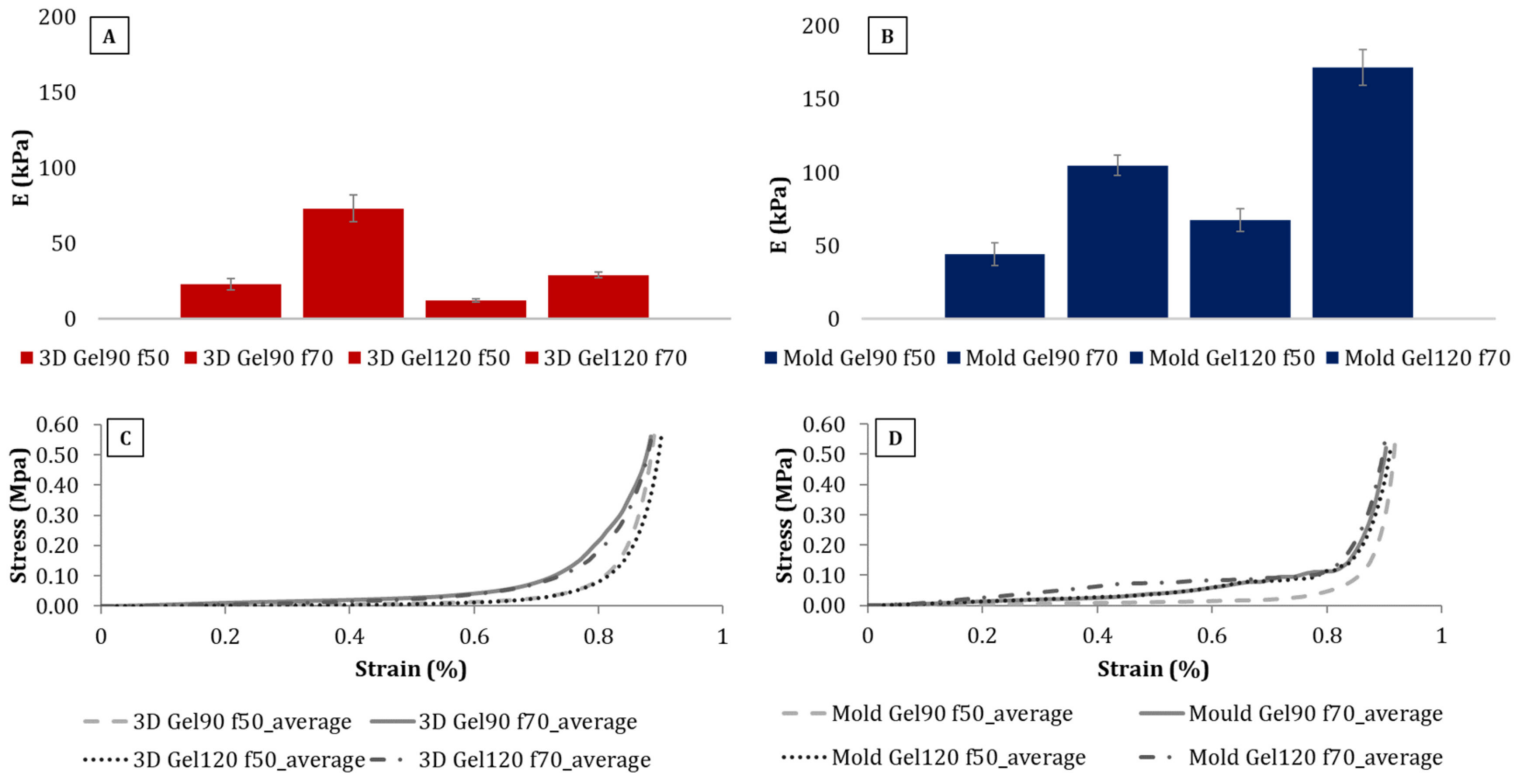
Young’s modulus and stress–strain curves of 3D printed (**A**,**C**) and mould casted (**B**,**D**) scaffolds.

Since the last aim of a biomaterial is to be implanted in vivo, testing its mechanical properties in in vivo-like conditions become crucial to better understand and predict its performance. All the samples tested were pre-conditioned overnight in PBS at 37 °C and then tested in uniaxial submersion-compression mode at the same conditions. The Young moduli (E), determined as angular coefficient of the stress–strain response from 0 to 10% strain (*n* = 9 ± std. dev.), showed a clear improvement in the compression resistance of the materials for both the 3D-printed and the mould-casted samples moving from f50 to f70 [33,41]. The mould-casted ones generally demonstrated higher Young moduli values. The higher values of the 3D Gel90 f50 and f70 confirmed what was previously discussed during the morphological evaluation, with a behavior more similar to the mould-casted Gel90 f50 and f70 than to the 3D Gel120 f50 and f70, attributable to their minor shape maintenance and less defined geometry (Figure 7).

Considering the higher fidelity to the model and better morphology reached with the 3D Gel120 samples, for a further evaluation and comparison of the mechanical properties of the 3D-printed and mould-casted scaffolds we decided to focus on the Gel120 version of both.

**Figure 8:**
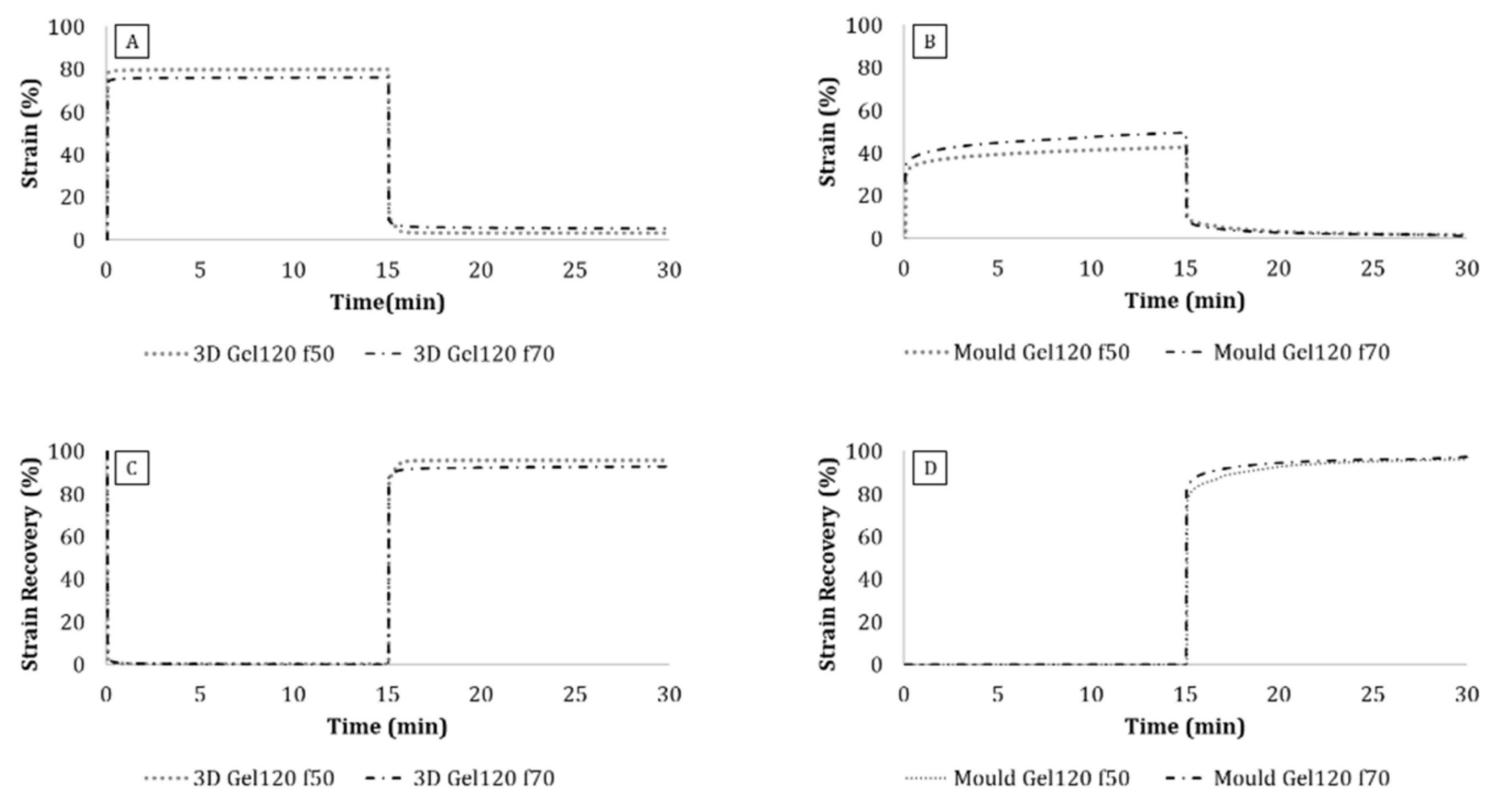
Compression resistance (**A**,**B**) and recovery ability (**C**,**D**) evaluation and comparison of the 3D-printed and mould-casted scaffolds.

Hence, the recovery ability of the scaffolds was then assessed by a creep test [33] performed setting a creep time of 15 min and a recovery time of 15 min with an applied stress of 6·10^−2^ MPa (Figure 8). It was clearly possible to see the higher tendency of the 3D-printed ones to quickly deform, reaching the 80% of deformation, as well as their extremely good recovery capability, of about 100% immediately after the compression force removal. The mould-casted ones showed, by contrast, a higher resistance to deformation, remaining under 60%, coupled with a slightly lower recovery ability, although still near to 100%.

**Figure 9:**
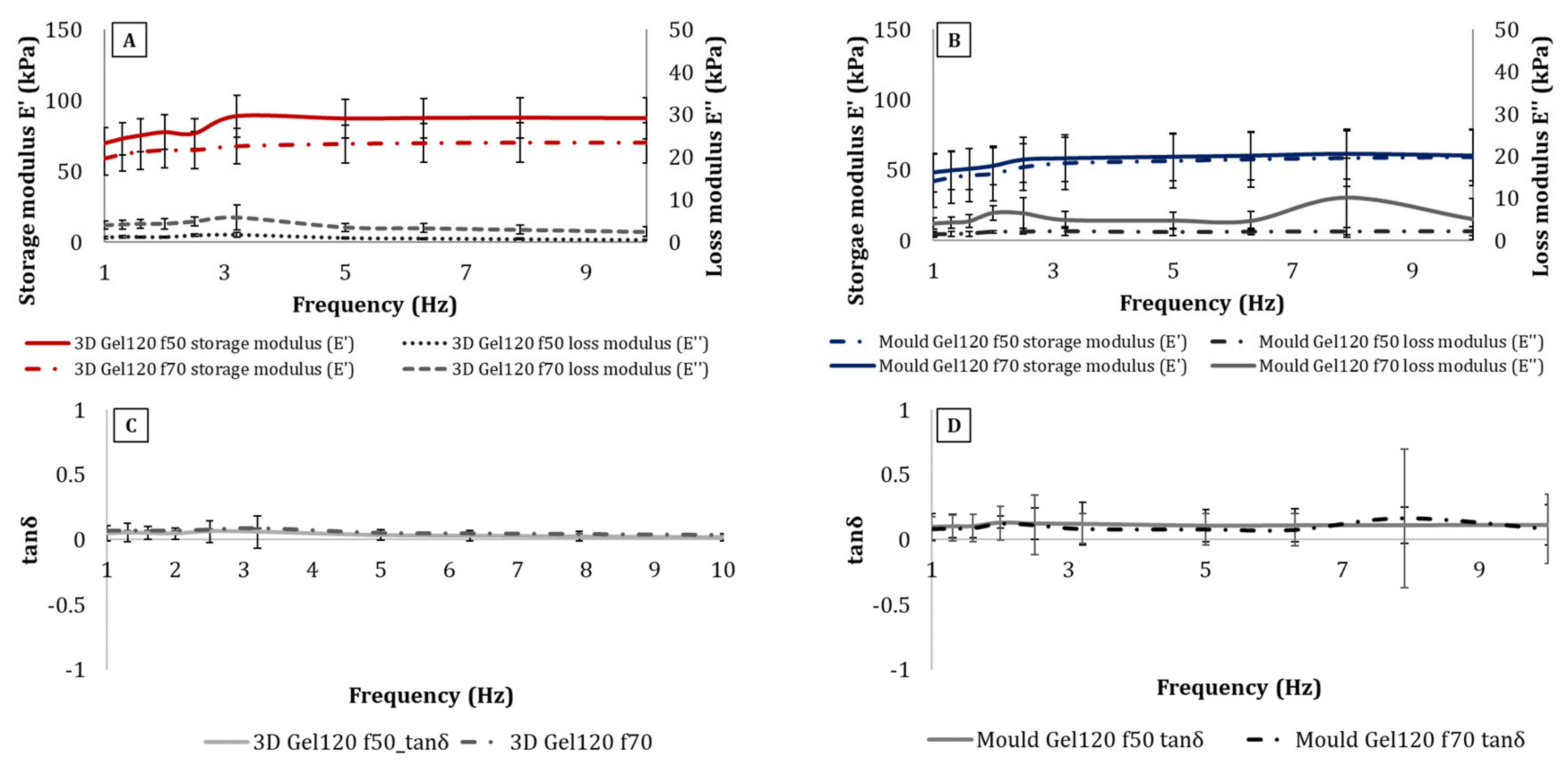
Evaluation of the storage modulus (**A**,**B**), loss modulus (**A**,**B**), and tanδ (**C**,**D**) of 3D-printed (**A**,**C**) and mould-casted (**B**,**D**) scaffolds through multifrequency test.

The viscoelastic behavior was finally determined as storage modulus E′ and loss modulus E″ by evaluating them through a multifrequency test [33] in a frequency range from 1 to 10 Hz. The loss modulus E″ resulted to be nearly not significant compared to the storage modulus E′, pointing out, as expected, the predominance of the elastic behavior for both the materials (Figure 9). Moreover, the higher values of the 3D-printed E′ compared to the mould-casted confirmed the propensity of this kind of scaffold to better withstand deformation, as already highlighted also by the creep test.

### 3.6. Biological Characterization

**Figure 10:**
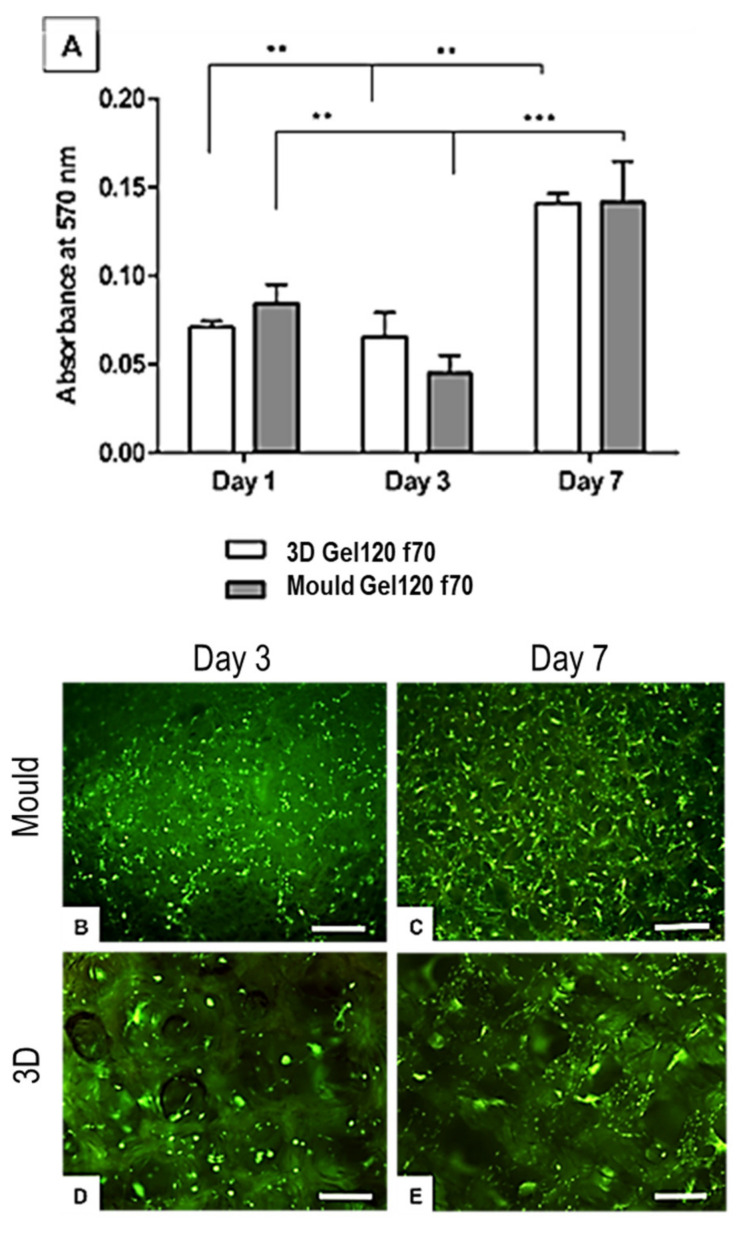
Cell viability analysis. MTT assay was performed after 1, 3, and 7 days of cell culture (**A**). The results were reported in the graph as mean ± standard error of the mean. ** *p*-value ≤ 0.01, *** *p*-value ≤ 0.001. Live&/Dead assay (Calcein stains live cells in green, ethidium homodimer-1 stains dead cells in red) on cells seeded on Gel120 f70 mould-casted (**B**) day 1; (**C**) day 3 and Gel120 f70 3D printed scaffolds (**D**) day 1, (**E**) day 3. Scale bars: 500 μm.

A preliminary study with human chondrocytes was performed to verify the suitability of both Gel120 f70 mould-casted and 3D-printed materials as scaffolds for cell colonization and viability. The in vitro investigation was performed on cell-seeded scaffolds, where cells were seeded onto the scaffold upper surface. Biological quantitative and qualitative tests on scaffolds were carried out by comparing the differential affection of the two material types on cell behavior. In detail MTT assay highlighted a statistically significant increase over the time of cell proliferation, demonstrating the absence of cytotoxic effect of both Gel120 f70-mould and 3D-printed scaffolds, with no differences between the two groups (Figure 10A). Confirming these positive results, the qualitative analysis of the upper surface by Live/Dead Assay of the scaffolds suggested that both materials were biocompatible and did not compromise cell viability (Figure 10B–E). In fact, a very high number of live cells were seen compared to a very low ratio of dead cells in both mould-casted and 3D-printed materials (Figure 10B–E).

**Figure 11:**
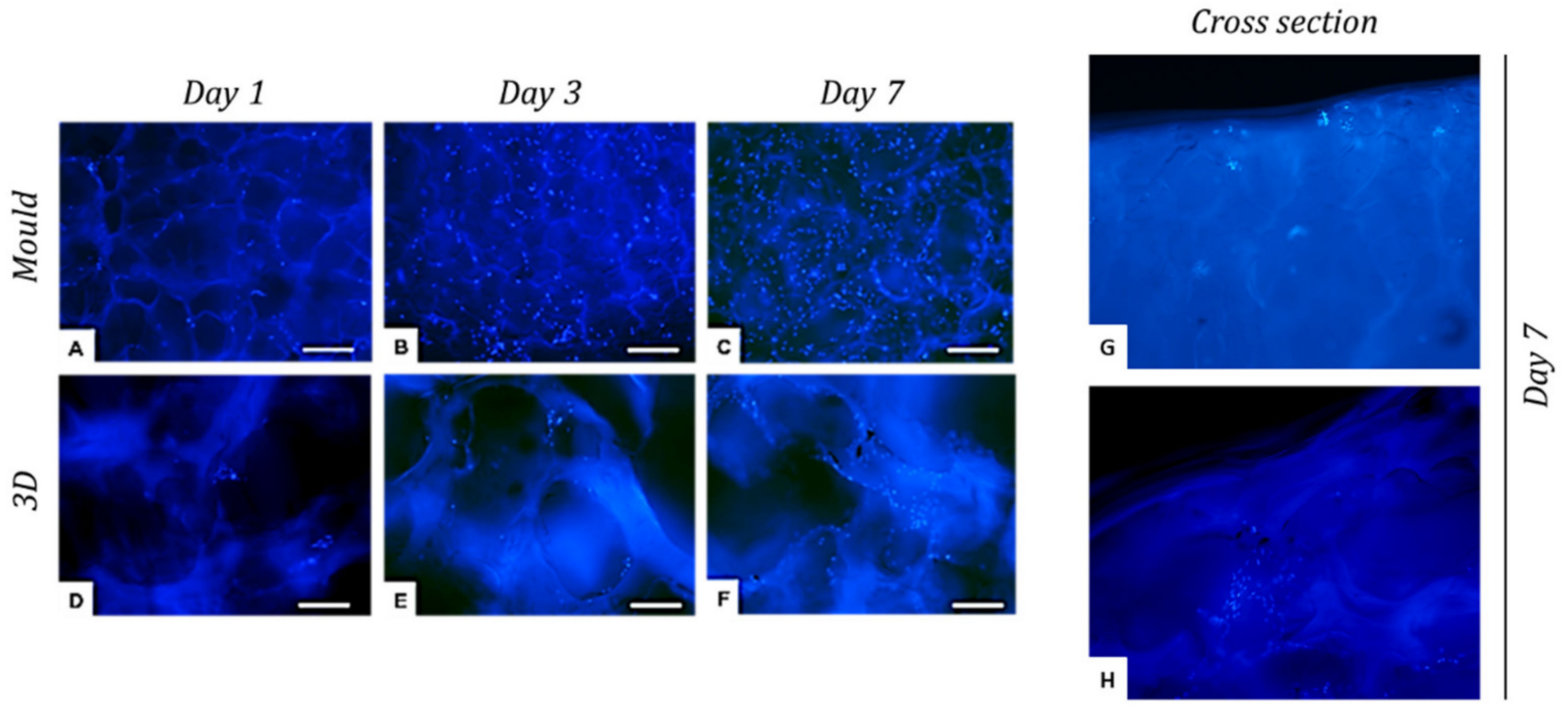
Scaffold colonization analysis by nuclei detection with DAPI in 3D-printed and mould-casted scaffolds at day 1 (**A**,**D**), day 3 (**B**,**E**), and day 7 (**C**,**F**). A cross section image was taken of both the scaffolds to highlight the inner spreading of the cells, respectively, within the mould-casted (**G**) and 3D-printed (**H**) scaffolds. Scale bars: (**A**–**F**) 200 µm; (**G**,**H**) 500 µm.

Moreover, the analysis of the upper surface by DAPI staining confirmed an increase in cell density over time in both the material types, despite the very different scaffold morphology (Figure 11A–F). In the Gel120 f70 mould-casted scaffold, the cells remained more anchored to the upper surface, creating a cell layer well visible at day 7 (Figure 11C,G). In the 3D-printed scaffold, by contrast, the cells penetrated deeper in the interstices of the sample, guaranteeing a better cell colonization of whole biomaterial, an index of a more real and mimetic attitude of in vivo situation (Figure 11F,H). This difference was confirmed by the analysis of the inner section of the two scaffolds: after 7 days the cells, seeded onto the upper surface, migrated through the porous structure and cells were seen to be well attached to the middle-lower level of the scaffold only in the 3D-printed scaffold, as shown by the section reconstruction in (Figure 11I,H).

## 4. Conclusions

A morphological, chemico-physical, mechanical, and biological properties evaluation of additive-free gelatin-based ink processed by 3D printing and mould casting was performed.

Interesting results from the 3D printing approach were recorded. A good printability and shape maintenance, proper geometry control in terms of alignment and model fidelity, no delamination and suitable medium uptake and degradation rate to allow an appropriate cell colonization and growth were discovered. For the mould casting, the morphology design turned out to be more difficult to lead, with the alignment ensuing as the only parameter possible to be actually controlled in the building process, through a thorough set-up of the freeze-casting process, and, partially, working on the Gel solution concentration. From the mechanical characterization, it was possible to infer the great ability of the 3D-printed scaffolds to face heavy deformations with complete recovery, contrarily to the higher resistance to deformation of the mould-casted ones. Cells’ spreading and attachment turned out to be less effective on the mould-casted scaffolds, despite the fact that they showed a good swelling ability and appropriate degradation rate for cell colonization, which is attributable to a smaller porosity, likely less interconnected, especially in the most concentrated ones. It was interesting to note how, starting from the same material and applying the same pre-process and post-process steps, the outcomes of the obtained scaffolds turned out to be significantly different and even diametrically opposed most of the time. The performed comparison highlighted the relevance of the shaping process when designing and realizing a device suitable for tissue engineering application, outlining another key parameter besides the starting material election. Depending on the features required to fulfil the chondral regenerative need, it was thus demonstrated that a different shaping process can be used to reach very distinct features. Nonetheless, we were able to print a collagen-derived polymer with no need of additive, nor for the printing nor for the crosslinking, showing a good biocompatibility combined with an eased cell colonization and spread, thanks to the better pore size and geometry control.

## Figures and Tables

**Table 1:** Gel solution concentrations for the realization, respectively, of the 3D-printed and mould-casted scaffolds. The mould casting solutions were adapted considering the 3D-printed scaffolds’ infill percentages.

	3D Printing		Mould Casting	
Code	Infill Rate	Solution Concentration	Infill Rate	Solution Concentration
Gel90 f50	50%	90 mg/mL	100%	45 mg/mL
Gel90 f70	70%		100%	63 mg/mL
Gel120 f50	50%	120 mg/mL	100%	60 mg/mL
Gel120 f70	70%		100%	84 mg/mL
Gel150 f50	50%	150 mg/mL	100%	75 mg/mL
Gel150 f70	70%		100%	105 mg/mL

## Data Availability

All data are contained within the article.

## References

[1] Gu L., Li T., Song X., Yang X., Li S., Chen L., Liu P., Gong X., Chen C., Sun L. (2021). Preparation and characterization of methacrylated gelatin/bacterial cellulose composite hydrogels for cartilage tissue engineering. Regen. Biomater..

[2] Jacob G., Shimomura K., Nakamura N. (2020). Osteochondral Injury, Management and Tissue Engineering Approaches. Front. Cell Dev. Biol..

[3] Jiang S., Guo W., Tian G., Luo X., Peng L., Liu S., Sui X., Guo Q., Li X. (2020). Clinical Application Status of Articular Cartilage Regeneration Techniques: Tissue-Engineered Cartilage Brings New Hope. Stem Cells Int..

[4] Tschon M., Brogini S., Parrilli A., Bertoldi S., Silini A., Parolini O., Faré S., Martini L., Veronesi F., Fini M. (2020). Assessment of the in vivo biofunctionality of a biomimetic hybrid scaffold for osteochondral tissue regeneration. Biotechnol. Bioeng..

[5] Turnbull G., Clarke J., Picard F., Riches P., Jia L., Han F., Li B., Shu W. (2018). 3D bioactive composite scaffolds for bone tissue engineering. Bioact. Mater..

[6] Abdulghani S., Mitchell G.R. (2019). Biomaterials for in situ tissue regeneration: A review. Biomolecules.

[7] Bobbert F.S.L., Zadpoor A.A. (2017). Effects of bone substitute architecture and surface properties on cell response, angiogenesis, and structure of new bone. J. Mater. Chem. B.

[8] Weber F.E. (2019). Reconsidering Osteoconduction in the Era of Additive Manufacturing. Tissue Eng.-Part B Rev..

[9] Bose S., Koski C., Vu A.A. (2020). Additive manufacturing of natural biopolymers and composites for bone tissue engineering. Mater. Horizons.

[10] Ligon S.C., Liska R., Stampfl J., Gurr M., Mülhaupt R. (2017). Polymers for 3D Printing and Customized Additive Manufacturing. Chem. Rev..

[11] Bello A.B., Kim D., Kim D., Park H., Lee S.H. (2020). Engineering and functionalization of gelatin biomaterials: From cell culture to medical applications. Tissue Eng.-Part B Rev..

[12] Bose S., Ke D., Sahasrabudhe H., Bandyopadhyay A. (2018). Additive manufacturing of biomaterials. Prog. Mater. Sci..

[13] Chun H.J., Park C.H., Kwon I.K., Khang G. (2018). Cutting-Edge Enabling Technologies for Regenerative Medicine.

[14] Li J., Wu C., Chu P.K., Gelinsky M. (2020). 3D printing of hydrogels: Rational design strategies and emerging biomedical applications. Mater. Sci. Eng. R Rep..

[15] Gong J., Schuurmans C.C.L., van Genderen A.M., Cao X., Li W., Cheng F., He J.J., López A., Huerta V., Manríquez J. (2020). Complexation-induced resolution enhancement of 3D-printed hydrogel constructs. Nat. Commun..

[16] Li J., Liu X., Crook J.M., Wallace G.G. (2020). 3D Printing of Cytocompatible Graphene/Alginate Scaffolds for Mimetic Tissue Constructs. Front. Bioeng. Biotechnol..

[17] Spencer A.R., Shirzaei Sani E., Soucy J.R., Corbet C.C., Primbetova A., Koppes R.A., Annabi N. (2019). Bioprinting of a Cell-Laden Conductive Hydrogel Composite. ACS Appl. Mater. Interfaces.

[18] Wang S., Hashemi S., Stratton S., Arinzeh T.L. (2021). The Effect of Physical Cues of Biomaterial Scaffolds on Stem Cell Behavior. Adv. Healthc. Mater..

[19] Li N., Qiao D., Zhao S., Lin Q., Zhang B., Xie F. (2021). 3D printing to innovate biopolymer materials for demanding applications: A review. Mater. Today Chem..

[20] Liu J., Sun L., Xu W., Wang Q., Yu S., Sun J. (2019). Current advances and future perspectives of 3D printing natural-derived biopolymers. Carbohydr. Polym..

[21] Russo T., D’Amora U., Gloria A., Tunesi M., Sandri M., Rodilossi S., Albani D., Forloni G., Giordano C., Cigada A. (2013). Systematic analysis of injectable materials and 3D rapid prototyped magnetic scaffolds: From CNS applications to soft and hard tissue repair/regeneration. Procedia Eng..

[22] Zhang F., Ma Y., Liao J., Breedveld V., Lively R.P. (2018). Solution-Based 3D Printing of Polymers of Intrinsic Microporosity. Macromol. Rapid Commun..

[23] Zhang B., Li S., Hingorani H., Serjouei A., Larush L., Pawar A.A., Goh W.H., Sakhaei A.H., Hashimoto M., Kowsari K. (2018). Highly stretchable hydrogels for UV curing based high-resolution multimaterial 3D printing. J. Mater. Chem. B.

[24] Kwon H., Paschos N.K., Hu J.C., Athanasiou K. (2016). Articular cartilage tissue engineering: The role of signaling molecules. Cell. Mol. Life Sci..

[25] Huey D.J., Hu J.C., Athanasiou K. (2012). Unlike bone, cartilage regeneration remains elusive. Science.

[26] Feliciano A.J., van Blitterswijk C., Moroni L., Baker M.B. (2021). Realizing tissue integration with supramolecular hydrogels. Acta Biomater..

[27] Holzapfel B.M., Reichert J.C., Schantz J.T., Gbureck U., Rackwitz L., Nöth U., Jakob F., Rudert M., Groll J., Hutmacher D.W. (2013). How smart do biomaterials need to be? A translational science and clinical point of view. Adv. Drug Deliv. Rev..

[28] Liu X., Holzwarth J.M., Ma P.X. (2012). Functionalized Synthetic Biodegradable Polymer Scaffolds for Tissue Engineering. Macromol. Biosci..

[29] Murphy C.A., Costa J.B., Silva-Correia J., Oliveira J.M., Reis R.L., Collins M.N. (2018). Biopolymers and polymers in the search of alternative treatments for meniscal regeneration: State of the art and future trends. Appl. Mater. Today.

[30] Owen S.C., Shoichet M.S. (2010). Design of three-dimensional biomimetic scaffolds. J. Biomed. Mater. Res.-Part A.

[31] Irastorza A., Zarandona I., Andonegi M., Guerrero P., de la Caba K. (2021). The versatility of collagen and chitosan: From food to biomedical applications. Food Hydrocoll..

[32] Panseri S., Montesi M., Dozio S.M., Savini E., Tampieri A., Sandri M. (2016). Biomimetic scaffold with aligned microporosity designed for dentin regeneration. Front. Bioeng. Biotechnol..

[33] Campodoni E., Heggset E.B., Rashad A., Ramírez-Rodríguez G.B., Mustafa K., Syverud K., Tampieri A., Sandri M. (2019). Polymeric 3D scaffolds for tissue regeneration: Evaluation of biopolymer nanocomposite reinforced with cellulose nanofibrils. Mater. Sci. Eng. C.

[34] Li X., Chen S., Li J., Wang X., Zhang J., Kawazoe N., Chen G. (2016). 3D culture of chondrocytes in gelatin hydrogels with different stiffness. Polymers.

[35] Wegst U.G.K., Schecter M., Donius A.E., Hunger P.M. (2010). Biomaterials by freeze casting. Philos. Trans. R. Soc. A Math. Phys. Eng. Sci..

[36] Li W.L., Lu K., Walz J.Y. (2012). Freeze casting of porous materials: Review of critical factors in microstructure evolution. Int. Mater. Rev..

[37] Chen X., Zhou L., Xu H., Yamamoto M., Shinoda M., Kishimoto M., Tanaka T., Yamane H. (2020). Effect of the application of a dehydrothermal treatment on the structure and the mechanical properties of collagen film. Materials.

[38] Krishnakumar G.S., Gostynska N., Dapporto M., Campodoni E., Montesi M., Panseri S., Tampieri A., Kon E., Marcacci M., Sprio S. (2018). Evaluation of different crosslinking agents on hybrid biomimetic collagen-hydroxyapatite composites for regenerative medicine. Int. J. Biol. Macromol..

[39] Shankar K.G., Gostynska N., Montesi M., Panseri S., Sprio S., Kon E., Marcacci M., Tampieri A., Sandri M. (2017). Investigation of different cross-linking approaches on 3D gelatin scaffolds for tissue engineering application: A comparative analysis. Int. J. Biol. Macromol..

[40] Franks F. (1998). Freeze-drying of bioproducts: Putting principles into practice. Eur. J. Pharm. Biopharm..

[41] Gostynska N., Shankar Krishnakumar G., Campodoni E., Panseri S., Montesi M., Sprio S., Kon E., Marcacci M., Tampieri A., Sandri M. (2017). 3D porous collagen scaffolds reinforced by glycation with ribose for tissue engineering application. Biomed. Mater..

[42] Sandri M., Filardo G., Kon E., Panseri S., Montesi M., Iafisco M., Savini E., Sprio S., Cunha C., Giavaresi G. (2016). Fabrication and pilot in vivo study of a collagen-BDDGE-elastin core-shell scaffold for tendon regeneration. Front. Bioeng. Biotechnol..

[43] Campodoni E., Dozio S.M., Panseri S., Montesi M., Tampieri A., Sandri M. (2020). Mimicking Natural Microenvironments: Design of 3D-Aligned Hybrid Scaffold for Dentin Regeneration. Front. Bioeng. Biotechnol..

[44] Campodoni E., Montanari M., Dozio S.M., Heggset E.B., Panseri S., Montesi M., Tampieri A., Syverud K., Sandri M. (2020). Blending gelatin and cellulose nanofibrils: Biocomposites with tunable degradability and mechanical behavior. Nanomaterials.

[45] O’donnell B.T., Al-Ghadban S., Ives C.J., L’ecuyer M.P., Monjure T.A., Romero-Lopez M., Li Z., Goodman S.B., Lin H., Tuan R.S. (2020). Adipose tissue-derived stem cells retain their adipocyte differentiation potential in three-dimensional hydrogels and bioreactors. Biomolecules.

[46] Le Guéhennec L., Van hede D., Plougonven E., Nolens G., Verlée B., De Pauw M.C., Lambert F. (2020). In vitro and in vivo biocompatibility of calcium-phosphate scaffolds three-dimensional printed by stereolithography for bone regeneration. J. Biomed. Mater. Res.-Part A.

[47] Wolf B., Lam S., Kirkland M., Frith W.J. (2007). Shear thickening of an emulsion stabilized with hydrophilic silica particles. J. Rheol..

[48] Ding H., Geng J., Lu Y., Zhao Y., Bai B. (2020). Impacts of crosslinker concentration on nanogel properties and enhanced oil recovery capability. Fuel.

[49] Van Krevelen D.W., Te Nijenhuis K. (2009). Typology of Properties. Properties of Polymers.

[50] Dhanumalayan E., Joshi G.M. (2018). Performance properties and applications of polytetrafluoroethylene (PTFE)—A review. Adv. Compos. Hybrid Mater..

